# The nature of AI: Metabolism, energy, water, labour and justice in the urban political ecology of artificial intelligence

**DOI:** 10.1177/30497515251344495

**Published:** 2025-05-30

**Authors:** Federico Cugurullo, Federico Caprotti, Jennie Day, Shona Geoghegan, Casey R Lynch, Filippo Menga, Caitlin Robinson, Joe Williams

**Affiliations:** 1 Department of Geography, 8809 Trinity College Dublin, Dublin, Ireland; 2 Department of Geography, 3286 University of Exeter, Exeter, UK; 3 Department of Criminology, 6363 University of Ottawa, Ottawa, Canada; 4 Department of Geography, 16738 University of Girona, Girona, Spain; 5 Dipartimento di Lingue, Letterature e Culture Straniere, 18953 University of Bergamo, Bergamo, Italy; 6 School of Geographical Sciences, 1980 University of Bristol, Bristol, UK; 7 School of Geography and Planning, 2112 Cardiff University, Cardiff, UK

**Keywords:** artificial intelligence, urban political ecology, urban AI, urban metabolism, justice, urban futures

## Abstract

The integration of vast volumes of Artificial Intelligence (AI) technology into the built environment is changing the metabolism of urban spaces. Due to the presence of various AIs in urban systems, there are now more agentic forces influencing the trajectory of urban development and entangling with pre-existing biological intelligences. Because of AI's substantial environmental costs, more resources are now needed to satisfy cities’ technological appetite. Urban futures are also becoming more uncertain as private AI companies gain considerable power in urban governance through oligarchic schemes that leave citizens with no voice. In this paper, we bridge Urban Political Ecology (UPE) and urban AI literature, in order to critically examine the nature of AI as it intertwines with urban living and urban infrastructure. More specifically, we offer a threefold contribution to knowledge. First, we examine how the advent of urban AI is altering urban metabolism, zooming in on specific socio-environmental issues pertaining to energy, water and labour. Second, we discuss how the urban metabolisms altered by AI are reproducing uneven dynamics of development that are ultimately leading to different forms of injustice. Third and finally, we propose a potential course of action to politicize urban AI and intervene on its evolution.

## Introduction

The present and future of cities are increasingly intertwining with the development of Artificial Intelligence (AI). There is a substantial body of evidence showing how, across spaces and scales, many different AIs are being integrated into urban systems (Cugurullo, 2021; While et al., 2021; Yigitcanlar et al., 2024). As a result, the integration of vast volumes of AI technology into the built environment is changing the metabolism of urban spaces, in complex ways that remain largely unexplored. In this context, we argue that Urban Political Ecology (UPE) can offer important theoretical and analytical perspectives to shed light on the urban transformations triggered by AI and, above all, on their socio-environmental implications.

Thematically, this paper focuses on *urban AI* defined as a heterogenous set of AI technologies “capable of acquiring and making sense of information on the surrounding urban environment, eventually using the acquired knowledge to act” in and onto urban spaces in an *autonomous* manner, in the sense that humans are often neither guiding nor supervising their actions (Cugurullo, 2020: 3). Real-life examples of urban AI include autonomous vehicles (AVs) mediating the transport portfolio of a growing number of cities; robots and drones operating a wide range of urban services, such as health, education, security, retail and waste management; city brains in charge of entire domains of urban governance; and invisible yet deeply agentic software agents that take fundamental decisions in the life of citizens, identifying for example who will get access to a mortgage or an health insurance and whom will be targeted by the police due to hypothetical criminal activities foreseen via predictive algorithms (Acheampong et al., 2021; Cugurullo et al., 2023; While et al., 2021; Xu et al., 2024).

In this context, the autonomy that urban AI is gaining when it acts in urban environments in an unsupervised manner, as well as when it mediates and influences urban governance, should not make us oblivious to the role that powerful human stakeholders play in its diffusion. In fact, “political decisions made by humans place urban AIs in the position to make decisions about the governance of cities” (Cugurullo et al., 2024: 1175). These include national AI strategies whereby politicians invest in AI technology and in large volumes of infrastructure meant to accelerate its deployment; and consortia of public and private actors, such as city councils, planning departments and tech companies, that join forces at the local level to co-develop and integrate AI into the built environment, in a manner that resonates with long-standing smart-city initiatives (Bareis and Katzenbach, 2022; Cugurullo, 2024; Zhang et al., 2022).

A focus on urban AI combined with an UPE perspective is helpful to realize how, with the advent of AI, the life and development of cities are becoming more and more complicated and puzzling. Due to the presence of various AIs in urban systems, there are now more agentic forces influencing the trajectory of urban development and entangling with pre-existing biological intelligences. Because of AI's substantial environmental costs, more resources are now needed to satisfy cities’ technological appetite. Urban futures are also becoming more uncertain as private AI companies gain considerable power in urban governance through oligarchic schemes that leave citizens with no voice. In other words, the plot thickens.

In addition, an UPE perspective can be helpful to critically examine and understand AI itself. While mainstream discourses present AI technologies as being *artificial* and *intelligent*, critical scholars stress that AIs are the product of human-made political economies and human labour, and that their autonomous actions rely on data produced by humans and thus reflecting human experiences (Bareis and Katzenbach, 2022; Cugurullo et al., 2024; Pasquinelli, 2023). There is therefore a fundamental connection between the socio-political dimension of AI and its technological apparatus which influence each other dialectically, as emblematically shown in the case of urban AI, since AI influences cities as much as the city influences AIs through subtle processes of co-constitution (Cugurullo et al., 2023). For a long time, UPE scholars have studied and conceptualized the development of cities and technologies in a dialectical manner, with an emphasis on how processes of techno-urban development become intrinsically connected with processes of capital accumulation which are, in turn, connected to processes of social and environmental exploitation (Connolly, 2019; Gandy, 2004; Kaika et al., 2023). Furthermore, UPE literature has stressed that such processes often take place in post-political contexts characterized by depoliticizing techno-managerial approaches meant to suppress any meaningful democratic action (Swyngedouw, 2018; Swyngedouw and Wilson, 2014). UPE is therefore a crucial lens to explore in theory and practice the evolving urban scenarios shaped by urban AI, so to better understand not simply how the plot is thickening, as the life of cities and their metabolism become more complex due to the integration of numerous AI technologies, but also what could be done to steer the urbanization of AI toward more emancipatory and democratic ends. In this sense, we mobilize UPE literature to call for a political engagement with urban AI discourses and practices, in an attempt to pre-empt the establishment of environmentally unsustainable and socially unjust manifestations of AI in the urban environment.

Through this paper, we bridge UPE and urban AI literature to critically examine the nature of AI as it intertwines with urban living and urban infrastructure. We refer to the notion of *nature* in several manners, by focusing on the fundamental character and qualities of AI, with an emphasis on its materiality. This technology is not pure and immaterial software. Instead, it is part of complex socio-environmental imbroglios with very tangible aspects that become particularly visible in the urban. This happens, for instance, when significant quantities of energy and water are consumed to sustain AI, often in and through large infrastructures located in peripheral urban spaces, such as data centres; when human labourers toil to maintain the production of AI and its training, in factories or remotely; and when AI gets integrated into the fabric of cities, and unevenly alters the life of citizens, by exploiting and excluding segments of the urban population. In addition, we refer to nature in an UPE fashion, by showing how, from a conceptual point of view, AI is indeed artificial in the sense that it resides in artefacts, including robots, AVs and urban infrastructures, but it is also natural since its very existence depends upon human lives, human-made decisions and natural resources, as part of complex socio-environmental entanglements where the boundaries between what is natural and what is artificial are fluid.

More specifically, we seek to accomplish three interconnected aims which mirror the structure of our contribution. First, we examine how the advent of urban AI is altering urban metabolism, zooming in on specific socio-environmental issues pertaining to energy, water and labour. Second, we discuss how the urban metabolisms altered by AI are reproducing uneven dynamics of development that are ultimately leading to different forms of injustice. Third and finally, we propose a potential course of action to politicize urban AI and intervene on its evolution. We do so by drawing upon a wide range of UPE conceptual perspectives and by proposing a series of areas of future empirical and theoretical research meant to pave the way for the next generation of UPE and AI scholars.

## Urban metabolism and urban AI

The rise of urban AI leads us to argue that the impact of AI on the city needs to be investigated in terms of its metabolic nature. Urban political ecology has, for many decades, featured analyses of the ways in which the city, technologies, infrastructures, people, bodies and elements of the natural environment are deeply interconnected and interrelated in the metabolic process of urbanization (Andueza et al., 2021; Tzaninis et al., 2021). Originally, much of the work on urban metabolism was interested in getting to grips with flows of materials, energy and resources into and out of the city, and in understanding the metabolic rift between the city and ‘Nature’. Much of this research grappled with the implicit (or sometimes explicit) question of whether the city specifically, and industrial capitalism more generally, and its impact on the natural environment was deleterious (Moore, 2000). Nonetheless, urban political ecologists have over the past three decades increasingly explored the multiple ways in which ‘Nature’ gets enrolled in material and political projects that are, in and of themselves, metabolic at heart (Gandy, 2018; Napoletano et al., 2015). This has helped academic debates to move past facile, modern binaries and dualisms that tended to posit what is ‘natural’ outside of the rational, societal sphere (Latour, 2012), and where capitalism could become reified as a set of extractive and exploitative processes that have agency on an otherwise passive and pristine ‘Nature’, in the process of destructive creation (Berman, 1982).

Where does urban AI fit into all of this? We offer four points for reflection and further enquiry. First of all, we stress that AI and the urban are both deeply intermeshed and exist in a dialectical relationship. Positing a (false) distinction between a disembodied notion of AI, the city, and nature risks recreating and retrenching binaries that are similar to the nature-city binaries mentioned above. In the case of urban AI, however, these binaries are represented by notions of AI as diffuse, immaterial and well-nigh invisible. Rather, urban political ecology-informed analyses of urban AI can do much to help unpack and critically analyse the ways in which AI is rooted in the materiality of the city, and of global urban networks that stretch from urban governance systems, specific technologies, energy sources, data centres, and networks of cables, communications, international standards, the urban knowledge economy, and legal, regulatory and ethical frameworks. These are part and parcel of the socio-natural urbanisation processes that now include urban AI.

Secondly, and linked to the point above, it is key to excavate and understand the ways in which nature, the city, and urban AI intersect in the production and reproduction of specific materialities and flows. In a way, this is not new: urban political ecologists have been unearthing the complex socio-natural relationship between the city and nature for a long time. However, given the deep impacts that urban AI is already having on contemporary cities and their cultures, economies, governance and polities, it is crucial to integrate insights from UPE into studies of how urban AI and nature co-produce the urban. Much promising work has been done on specific aspects linked to this theme, in relation for example to the political ecology of data (Nost and Goldstein, 2022), data centres and their energy usage (Colona, 2023) and their links to security (Hunter, 2025), and other topics such as the design of AI tech (Palmini and Cugurullo, 2024). Turnbull et al. (2023), for instance, argue for a consideration of digital ecologies as a way of understanding digitally-mediated relationships between human and non-human elements. We believe that work in this vein can be usefully leveraged to move towards understanding the political ecology of urban AI.

Third, there are methodological and epistemological questions around how to understand the visualities of urban AI in ways that make it possible to ground urban political ecology analyses of the phenomenon. Here, we understand AI visualities as the set of practices through which AI is seen, known and perceived, and through which AI sees, knows and perceives considering its techno-social and adaptive capabilities. In this regard, we draw inspiration from the varied ways in which previous work has sought to understand socio-environmental and other urban processes as kaleidoscopic (Colona, 2023), as a complex *polyopticon* that “functions more like ecological intelligence than a human brain” (Sherman, 2023: 1215), or as visible/invisible not only to citizens, but in some cases to AI itself (Tironi and Rivera Lisboa, 2023). Engaging with the notion of urban AI visualities helps us, as urban scholars, to understand and then critically interrogate the visualities produced *about*, *by*, and *around* urban AI. Among other things, such an endeavour might then yield useful avenues for producing countervisualities (Gabrielson, 2020) of urban AI.

Finally, we echo but also question much of the recent literature on urban political ecology and urban metabolism, that underlines the potentially emancipatory pathways offered through notions of broadening social struggles and right to the city movements through the mobilisation of Lefebvre's concept of *autogestion*. In its narrowest sense, autogestion refers to control over socio-economic and political systems by those most affected by their workings, such as workers and urban citizens (Butler, 2023). And yet, as Napoletano et al. (2023) show, and as underlined by Butler (2023), it is possible to think of autogestion not only as control over the socio-economic sphere, but as a form of management and steering of techno-capitalist and state-led modes of domination of nature and the environment. The stated promise of urban AI is one of autonomous systems, including AI-driven Internet of Things (IoT) urban networks (Cugurullo et al., 2024). Many of these networks are influenced by, and dependent on, complex corporate-state configurations that risk precluding the potential of autogestion, or even an approach towards an emancipatory politics of urban AI. One of the challenges raised by the emergence of urban AI, therefore, is how to find space, within work on urban political ecology, for identifying emancipatory potential in contemporary iterations of urban AI.

## Energy and urban AI

Tracing the connections between energy and AI is an important way for UPE scholars to interrogate the materiality of urban AI. In general, energy is a focus of political ecology research because of its centrality to the materiality of political economy, culture, society and the exercise of political power. Indeed, energy is often seen as “a material basis of politics more broadly – underpinning such powerful concepts as modernity, democracy, and freedom” (Huber, 2015: 481). UPE scholars, specifically, have variously traced the connections between energy and urbanisation on themes including (but by no means limited to) the role of cities and fossil energy in the historical development of capitalism (Malm, 2016); the ways in which networked energy infrastructure reflects and reinforces urban inequalities (Silver, 2015; Verdeil, 2019); the metabolism of different flows of energy through urban space (Demaria and Schindler, 2016; Newell and Cousins, 2015); the energy infrastructures that connect cities to far-flung spaces through extended urbanisation (Marks and Zhang, 2019; Williams et al., 2020); the role of energy in the financialisation of urban space (Knuth, 2019); and of course the position of cities as both major drivers of climate change and spaces vulnerable to climate change (Broto and Bulkeley, 2013).

AI is often discussed in ways that obfuscate its materiality, but by examining the energy basis of urban AI we are faced inescapably with its tangibility, rooted as it is in real ecologies and flows (Monserrate, 2022). As Bourzac (2024: no page) notes, “computing might seem abstract, but there are physical forces at work,” adding that “any time that electrons move through chips, some energy is dissipated as heat.” In this section, we unpack these materialities along three lines: the use of energy by urban AI systems; the use of urban AI in energy systems; and the ways in which energy systems are shaping and limiting the scope of urban AI. Overall, in line with the previous section, we identify a dialectical relationship at play which becomes evident in the co-transformations of urban energy systems and AI systems.

One of the major areas of concern for UPE scholars is how AI will shape urban energy transitions, broadly understood as how socio-technical energy regimes become established, transform and decline over time and space (Boateng et al., 2023; Bouzarovski, 2022; Bridge et al., 2013), and what this means for energy justice and inequality. In debates over energy and urban AI, we tend to encounter two narratives. One that says AI will increase energy use efficiency, the other that stresses the increased energy demand resulting from AI algorithms and technologies. Certainly, the rapidity of the emergence of AI has become a major challenge for urban energy transitions because its applications, such as AVs, robots and drones, IoT, software and apps, as well as the data processing (Cugurullo et al., 2023; Luccioni et al., 2025), require energy. Often lots of energy.

Perhaps the most widely discussed of these has been data processing. According to the International Energy Agency, electricity demand from data centres is predicted to rise rapidly from 460 terrawatt hours (TWh) in 2022 (almost 2% of global demand) to 1000 TWh in 2026 (IEA, 2024). Although differentiating the specific contribution of AI (compared to other data processing and crypto currencies) is complex, it is clear that AI is now the major driver of growth in the energy requirements of data processing. AI search engine queries require ten times more energy than traditional Google searches (Coskun, 2024), and although there is variation between different models, generative tasks require orders of magnitude more energy than traditional task-specific data processing, with those involving images and video most energy-intense of all (Luccioni et al., 2024).^1^ The dominant model for most of the major AI companies has been to pursue exponential growth in data processing capacity, which is driving the corresponding growth of AI's energy footprint. Many commentators have referenced *Jevons’ paradox*, a concept from nineteenth century studies of how increased energy efficiency of technology was accompanied by rising, rather than declining, coal consumption (Polimeni and Polimeni, 2006). The particular energy intensity of US-led model of AI came under further scrutiny in early 2025 with the emergence of DeepSeek-R1, launched by the Chinese company DeepSeek. DeepSeek-R1 achieves similar outcomes to dominant large language models (LLM) systems like OpenAI's GPT, using training models that require a fraction of the computational power, and need between 10 and 40 times less energy (IER, 2025).

There is a particular urban geography to the energy intensity of AI. For example, Ireland has become a focus of growth in the sector in Europe because of its favourable tax conditions. Data centres, almost all of which are located in and around Dublin (DataCenterMap, 2025), could account for 32% of the country's electricity demand by 2026 (IEA, 2024). Similarly, electricity use by data centres in Denmark, most of which are in Copenhagen, could rise to 20% of total consumption (IEA, 2024). AI is therefore bound up with the urbanisation of energy both within the city (causing rising consumption, adding extra capacity on the grid, and requiring new infrastructures like charging/docking points), and as a planetary process because of the ways in which cities and non-cities are connected through energy infrastructure, including pipelines, sites of extraction like lithium and coal mines, hydropower dams, cables, emissions and toxic waste (see Marks and Zhang, 2019; Williams et al., 2020). The energy transformations necessitated by AI are driving rapid reorientation and urbanisation of energy, and often lead to new urban fractures and inequalities (Levenda and Mahmoudi, 2019).

Just as urban AI systems are making unprecedented demands for energy, the use of AI within urban energy systems is rapidly changing. In particular, AI is central to the ongoing digitalisation of energy systems and, in turn, is often positioned as a means of addressing concerns about energy security and net zero transitions (e.g., UK Parliament, 2024). Digitalisation creates a deluge of data that has the potential to offer insight into (and therefore efficiencies in) almost every aspect of complex energy systems, including generation, demand, transmission, storage, markets, and consumption, in near real-time (Sareen and Müller, 2023). However, only AI can make sense of data in such volumes. As grids evolve, from large power plants supplying homes to more distributed supply and small-scale production, information exchange and data processing of sufficient magnitude will become integral to make sense of these increasingly multi-directional flows (Rozite et al., 2023).

The potential useful applications of AI, however, are not a panacea for energy systems characterized by inequalities and injustices. For example, as AI is used in the development of energy markets and trading it will drive ongoing processes of financialisation of energy, and the various forms of dispossession and injustice these entail (see Knuth, 2023). The use of AI in urban energy systems also raises challenging questions around democratic governance and the urban commons; themes that have long been central to political ecology research (Turner, 2017). Given how critical energy infrastructures are to almost every aspect of urban life, and how central AI has become in energy networks, there is a need for trust in the algorithms used and the data on which these algorithms are trained, and therefore scrutiny and public participation. Such concerns have led the Linux Foundation to argue that because energy is such a critical infrastructure, it necessitates the use of open-source AI models (LF Energy, 2025).

Finally, energy systems are likely to shape the future scope and trajectory of urban AI. As Caprotti et al. (2024) note, AI works through and is shaped by existing systems. This is an important point in large technical systems like energy networks, which are characterised by path dependencies and huge inertias. Energy systems are made up of layered technologies and built environments, some components of which last for decades or centuries. Research on decay, maintenance and repair (some of which comes from UPE) shows how infrastructure systems do not operate seamlessly, but are often fragile and kept running through improvisation, incremental work and contingent human labour (De Coss-Corzo, 2021; Ramakrishnan et al., 2021). Such perspectives offer a counterweight to the kinds of utopianism we often see around urban AI. A lot of the techno-triumphalist narrative around AI is about making urban systems fully autonomous, but this is unlikely to be a smooth process in energy systems, which are complex assemblages of all manner of material, cultural, institutional and economic ‘acting’ (Bennett, 2005; Cugurullo, 2020). The messy realities of energy systems, therefore, will shape what is possible with urban AI in the future.

## Water and urban AI

Water has always played a central role in urban political ecology, and the reasons for this are clear. What better resource than water, which is at the same time unruly, vital and natural, yet also manageable, material and transient, to capture the multifaceted ecological relations unfolding in any urban space? Indeed, the term UPE was initially used by Swyngedouw (1996) in a study in which he used a cup of water as a lens to understand the political ecology of the urbanization process. As urban water systems started to expand in nineteenth-century industrial cities, writes Gandy (2004: 373), water was “a brutal delineator of social power which has at various times worked to either foster greater urban cohesion or generate new forms of political conflict”. Just as water has been central to political ecology, it has similarly shaped urban political ecology, and many of the early works on the subject can be easily ascribed to both fields (Gandy, 2002; Kaika, 2003; Keil, 2005; Robbins, 2007; Swyngedouw, 1997).

Given the current technological and urban trends discussed in the introduction, we can safely assume that urban AI will occupy the political and scholarly debate for many years and, as UPE scholars, we posit that water will be part of this. Anticipating the key challenges and opportunities these trends will bring is both exciting and complex. It is exciting, because for better or for worse, debates on AI have yet to show that sort of intellectual fatigue that inevitably manifests itself when we get excited about something else. And it is for the same reason that the task is also challenging. In spite of the excitement about some of the promises of AI (particularly in medical research or autonomous driving) AI will remain deeply embedded in human social, political, economic, and cultural structures, including those that underpin flows of water. Rather than viewing AI as a standalone force, we must understand it as yet another (albeit more sophisticated and autonomous) technology shaped by human intentions and agendas, and that, as such, will not necessarily lead to enhancements in efficiency or productivity. With this in mind, we outline a series of key issues that are likely to shape the relationship between water and urban AI in the years to come.

The first one concerns matters of privatization and control. As noted in UPE literature, the entanglement of the private and public sectors seems inescapable in all matters related to water (Menga et al., 2023; Menga, 2025). Rapid technological advancements are further exacerbating this trend. As cities adopt AI to enhance their water utilities, such as expensive, privately owned AI programs that predict pipeline failures, as is already the case in Tucson (Arizona) and Newark (New Jersey), questions arise about the further privatization of urban water management. How might this trend lead to increased corporate control over water resources? It is easy to imagine multinational corporations monopolizing AI-driven water technologies, undermining public accountability, and prioritizing profits over equitable distribution.

The second issue is about surveillance and data ethics. Once we acknowledge that private actors are playing an increasing role in water control (currently around one billion people receive their water from private companies), it becomes clear that those who control water also control its data, and the use of AI in water monitoring raises concerns about surveillance and data ethics. For instance, smart meters and AI systems that track individual and community water consumption could pose significant privacy risks. The data collected by these systems will be controlled by private entities and governments, making it vulnerable to breaches and exacerbating power imbalances at the expense of public interests. While this may not worry those who willingly share personal data with generative AI models, it could be a significant issue for individuals who, for various reasons, prefer to keep their information private.

Third, there is the question of environmental and resource ethics. As outlined above in relation to energy, AI relies heavily on data centres and computational power. Many of these use water-based cooling systems that add stress to already strained aquifers (Hogan, 2015; Luccioni et al., 2025). Just as with energy, each generative AI query consumes water and, overall, it is clear that AI systems and infrastructures require vast amounts of water, often in water-scarce urban areas competing with local populations.^2^ Recent research estimates that training GPT-3, for example, consumed approximately 700,000 litres of freshwater on-site for data centre cooling alone (Li et al., 2023). This figure underscores how the environmental footprint of AI extends beyond carbon emissions, implicating it in the material and infrastructural realities of water scarcity. As it is often the case with supposedly sustainable technologies and strategies, a tool promoted for sustainable water management can, paradoxically, contribute to broader environmental impacts that contradict its intended goals.

The fourth issue relates to algorithmic bias, infrastructure and maintenance inequalities. AI-driven water infrastructure, such as leak detection and predictive maintenance, can enhance efficiency. However, as mentioned earlier, behind every AI model is a human who trains it and these systems may prioritize maintenance in well-resourced areas, while neglecting aging or inadequate infrastructure in marginalized communities. Moreover, as AI adoption accelerates, its integration (often requiring significant financial and technical resources) risks widening the gap between cities and neighbourhoods with differing capacities.

Fifth, there is the long-standing issue of participation and justice. UPE scholarship has long emphasized the role of communities in shaping urban environments (Nolan et al., 2022; Radonic and Kelly-Richards, 2015). However, the increasing reliance on AI in water management risks marginalizing these voices by prioritizing technical expertise over participatory decision-making. This shift could side-line local communities in urban water planning, reducing their agency in shaping policies that directly affect them. Moreover, many AI models function as ‘black boxes,’ with an opaque decision-making process that is difficult for non-specialists to interpret (Cugurullo and Xu, 2025). This lack of transparency further limits meaningful community engagement in governance, reinforcing existing power imbalances.

Finally, we want to highlight geographical global North-South disparities. AI development is concentrated in the Global North, creating a technological dependency in which cities in the Global South rely on AI solutions designed elsewhere, often without consideration for their unique socio-environmental contexts. This dynamic raises concerns about data colonialism, where AI-driven water management systems collect and utilize data from the Global South without necessarily benefiting the communities they serve, thus replicating broader forms of infrastructural violence that often mark postcolonial relations (Nolan et al., 2020). Data generated locally may be extracted, processed, and monetized by external entities, reinforcing existing inequalities and limiting local control over critical water resources. From an UPE perspective, addressing these challenges involves exploring ways to promote transparency, accountability, and the incorporation of local knowledge and participatory approaches in AI-driven water governance.

## Labour and urban AI

Labour has long been an important theme of research in urban political ecology. Indeed, one of the underlying tenets of political ecology, broadly speaking, is the recognition that ecology and environmental flows are inseparable from political economy, including questions of power, property relations, economic production and consumption. Scholars of UPE have traced how systems for the transportation, treatment, and provision of water, for instance, and for the removal and treatment of wastewater rely on multiple forms of labour for their construction, maintenance and everyday operations (Radonic and Kelly-Richards, 2015). Where these systems are absent or inadequate, the provision of water may require other forms of physical and emotional labour often bore by women within overall responsibilities for social reproduction (Doshi, 2017). Likewise, numerous studies from an UPE perspective have considered the various, often differentiated (by race, gender, caste, etc.), forms of labour through which urban waste economies are organized and maintained (Moore, 2011; Ranganathan, 2022; Wittmer, 2023). There is an inherent recognition that the environment is at least in part the product of human labour, and as such that the conditions and organization of human labour matter to questions of ecology, and vice versa, in a dialectical relationship.

At the same time, critical scholars of AI have considered the multiple entanglements of AI and labour practices and economies, most directly in discussions around the replacement or displacement of human labour with AI-powered machines (Bissell and Del Casino, 2017; Clifton et al., 2020; Lynch et al., 2022; Walker and Winders, 2024). In relation to the emerging literature on urban AI specifically, there is often an implicit assumption that new AI systems entail re-organizations of urban labour. The widespread adoption of autonomous taxis or buses, or of delivery robots, would necessarily entail the displacement of human taxi drivers, bus drivers, and delivery workers (Pakusch et al., 2021), while city brains may reduce the need for traffic engineers and transportation planners (Caprotti and Liu, 2022; Xu et al., 2024).

Beyond the question of labour displacement, observers of AI also highlight the central role of labour to the ongoing production of AI systems themselves (Jones, 2021), as well as the use of AI to more tightly surveil, reorganize, and control labour (Gent, 2024; Pasquinelli, 2023). AI systems rely upon the continuous generation and extraction of urban data. Much of these data are extracted through the labour processes that maintain everyday urban systems and practices of consumption (Attoh et al., 2019; Huws, 2020). At the same time, consumers and urban denizens are integrated into extensive systems of data production and capture that enroll them in forms of unpaid labour producing value for digital technology companies (Ettlinger, 2019). These data feed algorithms that depend upon data workers to annotate, classify or filter content. These workers are often dispersed around the world, employed under precarious conditions, and face serious health and safety issues (Gray and Suri, 2019; Muldoon et al., 2024; Williams et al., 2022). The output of AI systems may then be integrated into yet other labour processes to reorganize the division of labour or manage, surveil, and evaluate workers with the goal of increasing efficiencies to extract value (Gent, 2024). The centrality of workers and the labour process in the functioning of AI systems is perhaps best summed up by Pasquinelli's (2023: 12) labour theory of automation, which approaches AI as a division of labour in which “collective knowledge and labour” are “the primary source of the very ‘intelligence’ that AI comes to extract, encode and commodify.”

Given the centrality of labour as a question of concern for both UPE and urban AI literature, we highlight five key themes and questions for future research. First, there is the question of infrastructural fragmentation. A key insight and contribution of UPE scholarship has been the recognition of the uneven and fractured nature of urban infrastructures and their relationship to issues of citizenship, inequality and injustice, with direct repercussions for the organization of both productive and socially-reproductive labour (Golub et al., 2013; McFarlane and Silver, 2017; Radonic and Kelly-Richards, 2015). As AI becomes increasingly integrated into basic urban infrastructures, it is likely that existing fragmentations and their implications will be exacerbated, especially as urban AI systems rely on and build upon existing systems and the availability of historical data. Labour is an important proxy lens through which to explore both the spatial and cognitive limits of AI-enabled systems. Exploring what human labour is required in relation to or in the absence of AI systems, and at different times and in different spaces, can help chart the splintered and uneven geographies of urban AI. At the same time, tracing the shifting conditions of labour may also highlight emerging frontiers of urban extraction, as previously disconnected urban areas or infrastructures become integrated into data-driven systems.

Second, divisions of urban labour represent a critical issue in the age of urban AI. We need to ask: Which of these jobs or tasks are being automated? Which new jobs or tasks are being created? Who carries out this work? How are different kinds of work variously valued or de-valued? And how are the social, spatial and economic relationships among these jobs organized? On the flipside, we also need to ask what kinds of labour become necessary in the absence or breakdown of an AI-driven urban system. Work on AI-driven labour automation more broadly has tended to highlight the ways ‘higher-level’ logistical and management work is taken over by advanced algorithms, while more manual forms of labour remain key to the functioning of systems. There is reason to believe this trend will continue in at least some cases of urban AI, such as trash collection, where logistics, route-planning and sorting may be AI-controlled (Ahmed et al., 2024), but human labour is still required on city streets and for certain manual tasks, at least for now. This labour, however, is already becoming the object of new forms of algorithmic management (Gent, 2024) and data extraction, potentially fueling future cycles of automation.

The third issue relates to matters of (post)human labour and multiple intelligences. The emerging AI-inflected divisions of labour discussed above raise key questions about the evolving agency of different more-than-human urban actors. UPE scholarship has recognized nature as an agential force alongside and entangled with human labour (Gabriel, 2014; Gareau, 2005), while writing by Amin and Thrift (2017), for instance, highlights the complex entanglements among human, animal, material and technological agencies. As AI systems take on an increasing number of tasks, it is imperative for scholars to trace the evolving assemblages of actors through which the work of producing, operating and maintaining urban systems is carried out. For example, recent research highlights the integration of deep learning algorithms into urban water management for tasks like “demand forecasting, leakage and contamination detection, sewer defect assessment, wastewater system state prediction, asset monitoring and urban flooding” (Fu et al., 2022: 1). While it is likely that human labour will remain vital to the functioning of such systems, we cannot ignore the significant agency of other non-human actors like water itself, the landscape, bacteria, the climate, or the various materials involved in plumbing systems. A political ecology of urban AI necessarily needs to account for this broad diversity of agencies, including the distinct cognitive and physical capacities of various human, digital and material actors (Lynch and Del Casino, 2020), and seek to understand how they interact and produce particular outcomes. UPE is well situated to help elucidate the entangled nature of these diverse agencies, such that labour and the human agency of workers is understood as co-constituted in relation to the agency exercised by both nature and emerging forms of AI.

Fourth, a political ecology of urban AI needs to account for the fundamental roles of both labour and the environment to the ongoing production of AI itself. As vital urban systems come to rely on AI, this relation necessarily extends to the broader infrastructures on which AI systems depend. Important work from UPE has already begun tracing some of these relations. For instance, Levenda and Mahmoudi (2019) explore the layered infrastructures, such as fiber optic cables and data centers, on which digital-mediated urban life relies, tracing the reliance on labour as well as water and energy resources in sites often far removed from the sites of digital consumption. Hypothetically, a system controlling trash collection in Paris or monitoring and responding to water demand in Barcelona may be reliant on the maintenance of a data center in Iceland or Denmark and the complex human and natural systems on which it depends. As Muldoon et al. (2024) have recently demonstrated, labour is a useful lens to trace the global relations through which AI is produced and maintained. Such a focus can also help highlight the broader metabolic flows which connect urban AI systems in a particular city to environments and economies in faraway places.

Fifth, labour organizing and political action constitute a key element in the contestation of urban futures, including their digital and ecological dimensions. Fredericks (2018), Demaria and Schindler (2016), and other UPE scholars have examined waste worker strikes as key forms of activism in which the normal flows of urban metabolism are intentionally disrupted in order to push for political, economic or social reforms. With the growing influence of AI, new forms of labour organizing are already emerging to confront threats to workers’ livelihoods, rights and autonomy. The Hollywood strikes of 2024, which included both actors and writers, have been held up as an example for future forms of AI-related labour activism, raising the prospect that collective bargaining could be employed as a strategy of localized AI governance (Ponce del Castillo, 2025). Likewise, the experiences (and successes) of platform worker organizing in places like Spain and Chile (Morales-Muñoz and Roca, 2022) or union organizing at Amazon (Delfanti, 2021) highlight the political agency of workers in shaping the integration of new technologies in everyday urban systems. Furthermore, movements toward alternative arrangements of labour such as cooperative and commons-based models of urban digitalization (see Grohmann, 2023; Lynch, 2020) point toward the possibilities for a more expansive political re-imagining of the possibilities for urban ecologies and the role of both human labour and AI within them.

## Justice and urban AI

The widespread integration of AI across urban landscapes and systems, as we illustrated above, raises legitimate questions around its impacts on justice in the city. As AI-driven systems continue to be implemented as a novel layer of urban infrastructures, the importance of adequately considering the ways in which these technologies alter everyday experiences of injustice continues to grow (Robin and Acuto, 2018). In this way, the longstanding emphasis UPE has placed on the materialization of (in)justice in the urban context (Heynen, 2003; Kaika and Swyngedouw, 2011; Keil, 2003; Swyngedouw and Heynen, 2003) provides an ideal framework for examining the novel processes and dynamics currently shaping our cities. Whilst UPE scholarship has traditionally focused on the implementation of critical urban infrastructure such as water, energy and waste facilities (Kaika and Swyngedouw, 2011; Swyngedouw and Heynen, 2003; Swyngedouw and Kaika, 2014), contemporary developments require an expanded focus on AI-driven technologies (Cugurullo et al., 2024; Nost and Goldstein, 2022; Schneider, 2019). This necessitates drawing on a broader array of knowledge systems, including those of frontline communities, global civil society actors, and the ethical tech movement, whose contributions often fall outside of traditional academic publication channels (Data and Society, 2025). With this in mind, as we confront the unfolding complexities of digital transformation in urban contexts, the tools of UPE can assist communities, policymakers, and scholars alike in ensuring justice characterises urban AI-powered governance.

From the outset of UPE as a field of academic inquiry, scholars have drawn on Marxian theories of development to critically analyse the processes through which urban environments are co-produced by a diverse array of actors with competing and often conflicting agendas (Swyngedouw and Heynen, 2003). As the Rawlsian ideals of distributive justice of the postwar era gave way to an entrepreneurial neoliberal paradigm, UPE provided a critical framework for examining how state, market, and ecological relations were reconfigured around individualistic, profit-driven imperatives (Swyngedouw and Kaika, 2003). In particular, this body of literature recognises asymmetrical power relations as fundamental structures which impact the distribution of resources and risks, thereby reproducing socio-spatial inequalities and reinforcing the uneven socio-environmental landscapes of urban life (Heynen, 2003; Keil, 2003; Swyngedouw and Heynen, 2003). The development and transformation of urban landscapes are not merely physical processes, but rather dynamic “socio-natures” (Swyngedouw, 2006: 109) shaped by the complex interplay of material, social, political, and cultural forces. The increasing integration of AI-driven technologies into these urban landscapes further complicates this interplay by redefining the mechanisms of governance and control (Cugurullo, 2021). As these technologies become deeply embedded within urban infrastructures, critical questions arise regarding who wields authority over AI-driven systems, how this control is enacted and whose interests are ultimately prioritised in shaping the future of urban environments (Cugurullo, 2020). In response, participatory algorithmic audits conducted by civil society groups have begun to emerge, such as those led by AlgorithmWatch (Marsh, 2024) or Mozilla's AI Intersections initiative (Rankin, 2024a), which illustrate an evolving power dynamic where civic actors are contesting and co-producing AI governance.

As previously established, the issue of uneven power dynamics within AI-driven infrastructures closely aligns with UPE's long-standing focus on how entrenched injustices in urban environments are either perpetuated or challenged. For example, the emerging debates surrounding the governance of digital infrastructure (Caprotti et al., 2024; Cugurullo and Xu, 2025; Kitchin, 2023) holds striking resemblance to earlier struggles around managing critical resources such as water and energy (Agyeman and Evans, 2003; Kaika and Swyngedouw, 2011; Swyngedouw and Kaika, 2014). In cities across the globe, the acts of capturing, processing and monetising data by private corporations has become increasingly commonplace, raising concerns about exploitation, exclusion, and heightened inequalities (Barns, 2024; Geoghegan and Cugurullo, 2025). When corporations control urban data, they possess the power to exploit citizen information for monetary gain which facilitates the reinforcement of profit-driven models that marginalise predominantly lower-income communities (Taylor, 2017). This has resulted in the emergence of movements, such as Data for Black Lives (2025) and Mozilla (2025), in support of open-source, community-led alternatives to proprietary AI systems. In a similar way to early UPE debates, the power relations embedded in these processes can be seen to exacerbate inequalities in a manner which mirrors the privatisation of essential resources like water and energy (Agyeman and Evans, 2003; Kaika and Swyngedouw, 2011; Swyngedouw and Kaika, 2014). As a result, the strive for citizen empowerment that characterised traditional UPE movements can be seen to translate seamlessly into contemporary times as the need for urban AI systems that serve all residents equitably continues to rise (Cuppini et al., 2025).

Secondly, the algorithmic mechanisms that underpin the decision-making processes of AI-powered technologies highlight the intricate interplay between hierarchical social structures and socio-technical systems within the urban landscape (Caprotti et al., 2024; Cugurullo et al., 2024). The datasets utilised by these systems for learning inherently embody societal biases, regulatory frameworks, and institutional priorities that exist within society more broadly (Calzada, 2019). These underlying influences can obscure or normalise discriminatory practices, ultimately shaping algorithms that lack neutrality and perpetuate systemic inequities (Knebel et al., 2022; Zuboff, 2019). In domains such as housing, policing, and health services, these biases can materialise in decisions that disproportionately disadvantage already marginalized communities (Cugurullo et al., 2024). Notably, the expansion of AI infrastructure has generated significant socio-environmental consequences for peripheral communities, particularly in areas surrounding large-scale data centers termed “sacrifice zones” (Bridges, 2024). This can be seen in the case of Brownsville, Texas, following the arrival of SpaceX where consequences have manifested in the form of community displacement, environmental pollution, and the intensification of extractive energy practices (Garcia et al., 2025). By highlighting the entanglements of power, space and technological infrastructures, UPE prompts critical questions about who benefits from AI-driven interventions in urban contexts and at whose expense. In doing so, it compels policymakers and stakeholders to work towards a future where AI deployments work toward equitable rather than exclusionary urban transformations.

Finally, the integration of AI-driven surveillance systems, including facial recognition, automated tracking, and predictive policing, greatly exacerbate existing power asymmetries in the city (Makanadar, 2024; Saheb, 2023; Zuboff, 2022). In line with the aforementioned issue of algorithmic bias, the opaque functionings of these technologies raise considerable concerns regarding justice and equality in the city (Cugurullo and Xu, 2025; Saheb, 2023). In particular, methods of predictive policing have received significant criticism as the reliance of such systems on historical crime data reinforce patterns of racial and socio-economic discrimination due to the frequent appearance of systemic bias in such datasets (Cugurullo et al., 2023). From an UPE perspective, the surveillant systems commonly found in urban AI infrastructure creates a new dimension to the governance of urban ecologies by further entrenching state control over public spaces (Baumann et al., 2024; Nost and Goldstein, 2022). Moreover, AI-driven surveillance systems exemplify the central concern in UPE scholarship that is the expansion of neoliberal governance whereby discourses of security and efficiency are leveraged to justify and reinforce deeply uneven power structures within the urban landscape. As a result, ethical tech practitioners, such the Mozilla team, have begun documenting how these surveillance systems disproportionately affect racialized (Rankin, 2024b) and disabled populations (Rankin, 2024c), adding an intersectional layer that UPE scholarship must increasingly engage with.

In light of the above, we argue that applying an UPE lens is crucial for illuminating the ways in which AI reconfigures power relations, resource allocation and equity in contemporary urban environments. The long history of UPE scholarship in working to uphold robust local governance and inclusive community participation (Kaika and Swyngedouw, 2011; Keil, 2003) serves as a crucial mechanism in the strive for equitable AI deployment. To fully realize this potential, however, UPE must incorporate the lived experiences, political strategies, and epistemic contributions of frontline communities and civil society organisations actively reshaping the digital city. Furthermore, as cities increasingly assert their influence on the global stage, municipal governments are positioned to effectively establish ethical AI standards, facilitate knowledge exchange, and champion equitable data governance both within and across borders (Calzada, 2018; Robin and Acuto, 2018). By leveraging international relations, municipal governments can advocate for ethical AI standards, equitable data governance, and share best practices across borders (Calzada, 2018; Robin and Acuto, 2018). Ultimately, enhancing local autonomy while forging strategic global partnerships positions cities as pivotal actors in shaping just and inclusive outcomes in the AI-driven urban future.

## Conclusions and ways forward

In this paper, we have drawn upon UPE perspectives to shed light on how the advent of urban AI is altering cities’ metabolism by impacting, for instance, on flows of energy, water and labour, in ways that risk penalizing already marginalized urban communities and further exploiting our ecosystems. This is a worrying scenario the demands not simply critical understandings but also political interventions, as our urban future becomes more and more intertwined with the future of AI in emerging *urban AI futures*. Such political interventions should be normalized because, as we have also shown in this paper, the nature of AI is partly our own nature, given that it is our labour, data, decisions and resources that shape and sustain its existence, particularly in urban environments.

However, the contemporary post-political landscape, characterised by depoliticizing techno-managerial approaches to public life that render emancipatory and democratizing actions impotent (Swyngdenouw, 2018), has negative implications for the emergence and trajectory of urban AI futures. Hegemonic discourse about urban AI futures is technologically determinist, suggesting that AI is a pre-determined and incontestable trajectory of development (Bissell, 2018; Halford and Southerton, 2024; Hopkins and Schwanen, 2021). In presenting urban AI as an inevitable technical issue rather than a site of democratic contention, political antagonism and debate is supressed and the depoliticized status quo is maintained (Swyngdenouw, 2018).

Urban AI futures are too important to remain depoliticized, outside of the realm of contestation, and in the hands of the minority political and economic elite. A groundswell of frontline communities and civil society groups are undertaking trailblazing activities to question, disrupt and resist AI in a way that resonates with Amoore's (2019) call for an *ethics of doubt*. In addition to exercising their right to doubt and oppose certain AI technologies, these groups are providing technological alternatives to advance equitable AI futures. For instance, movements such as ‘platform cooperativism’ seek to resist the impenetrability, complexity and extractive private ownership of “hyperlean digital platforms” (Moore and Bissell, 2024: 206). As an alternative, the movement presents visions of, and tangible technological interventions towards, building and implementing platforms that are cooperatively owned and managed by users. Entities such as Animikii are producing Indigenous Sovereignty Platforms that endeavour to collaborate with organisations to develop technology in a non-technologically solutionist, “culturally informed, respectful way” (Animikii, 2025: no page). Relatedly, the internal dissent within Big Tech continues to lead individuals in the ethical AI space, such as Meredith Whittaker and Timnit Gebru, to blow the whistle on problematic practices in the sector (Hicks, 2025). These former Google employees have gone on to lead Signal (an encrypted messaging communications platform) and the Distributed AI Research Institute, “a space for independent, community-rooted AI research, free from Big Tech's pervasive influence” respectively (DAIR, 2024: no page). Public resistance to urban AI is also increasingly prevalent. A notable example includes the resistance of San Francisco's citizens to the experimentation of autonomous robotic technologies in the city's public space (While, 2024), signalling a reassertion of antagonistic democracy and political struggle. Last but not least, art can be a powerful form of public resistance, as artists strive to cultivate alternative imaginations and aesthetics of AI across different spaces and cultures (Bridle, 2023). UPE academics must use their unique position to repoliticize the city through transformative democratic action, taking seriously the call to remember that “AI changes the nature of cities as much as the city changes the nature of AI, and we need to remind ourselves that we are the city” (Cugurullo et al., 2023: 383).

How UPE academics go about creating impact in their professional lives, individuals’ personal philosophies, worldviews and motivations that animate their professional contribution to public life, varies greatly and can be classified into a spectrum or simple typology of approaches. The first category can be understood as the pursuit of knowledge for knowledge's sake. It is the “search of the invisible so as to make it visible – and thus more mutable – to ourselves and to others” (Burawoy, 2022: 4); to interpret and understand. The second category can be considered resistance as activism, such as Swyngedouw's (2018) pivotal contributions to the UPE field that cultivate and inspire tactics of resistance, refusal, democratic debate and contention. Thirdly, the levers of policy and governance can represent a constructive form of intervention. The ‘policy academic’ is interested in a form of advocacy that engages critically with and formulates policy-oriented solutions to social problems (Burawoy, 2022). For instance, Kaika (2017) engages directly with policy debates and agendas about sustainability to critique narratives of resilience in smart cities. Authoring of briefing notes, policy recommendations or other policy-adjacent activities represent an effort to guide the direction of travel towards their normative socio-political predilections (Burawoy, 2022).

Evidently this typology is simplistic; we all likely straddle numerous categories and our approaches will ebb and flow throughout our careers. Notably, all approaches are valuable and necessary for excavating and interrogating the unfolding politics of urban AI before its technical and socio-material relations settle and solidify. However, Swyngedouw (2018: xvii) aptly warns that in contemporary post-political conditions, contentious issues can be “discussed, researched, dissected, evaluated, raised to issues of public concern, and debated at length in a variety of public and political arenas” and against our best wishes, our studious efforts spent examining and analysing can risk reifying the depoliticized status-quo. Therefore, a fourth underexplored, underleveraged and arguably controversial site of intervention presents a productive aperture for UPE scholars seeking to politicize urban AI. UPE scholars can and should go further than understanding the socio-political changes brought about by AI. Rather, transformative democratic political action necessitates repoliticization through action and intervention (Swyngedouw, 2018). Urban AI futures are too important to be left to the domains of economics or data science (Beckert, 2016; Halford and Southerton, 2024). Instead, UPE scholars are well placed to tangibly intervene to shape AI's direction of travel towards just socio-environmental urban futures.

Collaborating with AI engineers to translate ethico-political AI principles, policies and critical theories into applied engineering practice is a fruitful mechanism to bring about more socially just urban AI futures. This proposal is not new, of course. However, such an approach is presently under-utilized in UPE, to the detriment of the unfolding urban AI landscape. Our industrious identification of problematic AI logics can fall short of creating meaningful interventions if the engineering teams tasked with operationalising said policies do not have the tools to translate high level ethico-political concepts into their AI engineering operations on the ground (Millar et al., 2020). Even the most socially attuned AI policy will fall flat if AI engineers are unclear about how to implement it in practice. AI developers, those imagining, building and bringing AI futures into being, tend towards technosolutionist worldviews (Maalsen et al., 2024), neglecting to acknowledge that ‘perfect’ (i.e., technologically functional) technologies can fail in situ if they are not aligned with the values of their social, political and cultural contexts of application (Millar, 2020). While conceding the importance of ethical AI design, engineers frequently hold the view that AI professionals should stick to their silos: engineers should undertake AI engineering, and ‘miscellaneous others’ should undertake the qualitative, and therefore less pressing, ethics work. Such a view evidently falls short of realising that designing and developing AI technologies is a profoundly value-laden project (Millar, 2015).

The ideologically and professionally siloed AI ecosystem, with rampant ethical evasion in the present and deferral to the future (Day, 2023), is fertile ground for UPE scholars. We are not limited to empirically examining these dynamics and reporting our theorizations about the challenges they engender back to our narrow academic communities. Rather, we can close the knowledge translation loop by transporting these findings back to the tech space and translating their direct relevance and applicability to those with the power to shape the design of AI development. This collaborative, interdisciplinary and multi-sectoral work of bridging and translating work between ideological and professional silos necessitates a commitment of all parties (engineers and social scientists, or a wider multi-sectoral team) to genuine interdisciplinary partnership (Kitchin in Schechtner, 2017; Halford and Southerton, 2024). It requires the mutual cultivation of trust, respect for one another's operational expertise and goals together with a genuine curiosity about what lies beyond the confines of one's own worldview.

Accordingly, future urban AI UPE research directions or agendas exploring (metabolism, energy, water, labour and justice) relations could include multi-sector and multi-disciplinary teams of academic and professional AI software engineers, urban planners, civil engineers, social scientists, government officials, publics and beyond. For bridges to be built across such disparate clusters, it is crucial that deep understanding and empathy is developed for one another's perspectives, goals, constraints and values. This takes time, vulnerability and a desire to connect on a human level with colleagues beyond the strict confines of the practical task at hand. While ideological professional and values-based tensions will exist, collaborators should be united by a mutual desire to collaboratively shape ethical principles, governance frameworks, codes of practice and engineering design requirements that promote just AI futures. Many critical social scientists are understandably troubled by the unfolding AI landscape, and therefore may be reticent to be perceived as streamlining the unfolding of an AI project they are politically opposed to, in both a personal and professional capacities. However, given the exponential rate of change in contemporary AI development, the field of UPE has a unique window of opportunity to meaningfully intervene in the political shaping of urban AI futures prior to AI's crystallisation into our fragile urban ecosystems.

## Notes

1. According to Luccioni et al. (2024), task-specific functions require an average of between 0.002–0.007 kWh per 1,000 queries, while AI tasks consume between 0.038–2.902 kWh per 1,000 queries.

2. According to Lin et al. (2023: 2), GPT-3 consumes the equivalent of “a 500ml bottle of water for roughly 10–50 medium-length responses.”
